# Metastatic Papillary Thyroid Cancer to the Liver: The Central Role of a Multidisciplinary Approach to Treatment

**DOI:** 10.31486/toj.20.0067

**Published:** 2021

**Authors:** Adam I. Riker, Ian A. Hodgdon, Tracy A. Dewenter, Richard Marshall, Brian Boulmay

**Affiliations:** ^1^ Anne Arundel Medical Center, Geaton and JoAnn DeCesaris Cancer Institute, Department of Surgery, Cancer Service Line, Luminis Health, Annapolis, MD; ^2^ Department of Surgery, Louisiana State University Health Sciences Center, New Orleans, LA; ^3^ Department of Pathology, Louisiana State University Health Sciences Center, New Orleans, LA; ^4^ Department of Radiology, Louisiana State University Health Sciences Center, New Orleans, LA; ^5^ Section of Hematology/Oncology, Louisiana State University Health Sciences Center, New Orleans, LA

**Keywords:** *Lenvatinib*, *neoplasm metastasis*, *neoplasm recurrence–local*, *radiotherapy*, *thyroid cancer–papillary*, *yttrium-90*

## Abstract

**Background:** Differentiated thyroid cancer (DTC) is comprised of papillary and follicular subtypes, and both have an overall excellent long-term prognosis. Patients with localized DTC that is successfully treated, usually with surgery, exhibit long-term survival well above 90%. In contrast, patients who develop distant metastatic disease have a significantly worse overall prognosis and outcome, often with disease that is refractory to conventional therapy such as surgery, radioactive iodine, and hormone suppression. For patients who recur with distant metastatic disease, limited effective treatment options are available, and most die of their disease within 5 years of recurrence.

**Case Report:** We report the case of a 26-year-old female who presented with recurrent papillary thyroid cancer and a metastatic lesion isolated to the liver. Because of the extremely large size of the metastatic liver mass upon initial presentation, we took a neoadjuvant, multifaceted approach to treatment that included selective internal radioembolization therapy, an oral multikinase inhibitor, and surgical resection of the tumor mass after maximal reduction in tumor size. However, the patient died of metastatic DTC after 39 months of treatment.

**Conclusion:** A multimodal, comprehensive approach to managing such complex patients is essential to optimize both the sequence and therapeutic approach to treatment.

## INTRODUCTION

Approximately 90% of malignant thyroid tumors are well differentiated and are classified as papillary thyroid cancer (80%) or follicular carcinoma (10%).^1^ Together, they are referred to as differentiated thyroid cancer (DTC), which is relatively rare worldwide and accounts for <1% of all human cancers.^2^ Distant metastases occur during follow-up in 4% to 15% of patients.^3-5^ Even rarer are cases of DTC that metastasize only to the liver, with a reported incidence of <0.5% and <15 cases reported since 1970.^6-11^

In terms of multidisciplinary management, the treatment options may include radioactive iodine (RAI) therapy with iodine (I)-131, oral kinase inhibitors, local ablative approaches, and, rarely, surgical resection. Limited data are available on the optimal approach to disease management; the overall prognosis for patients with metastatic DTC is poor, and long-term survival ranges from 10% to 50% at 10 years.^10-14^

We present the case of a 26-year-old female who presented with a locoregional occurrence of papillary DTC and an isolated metastasis to the liver.

## CASE REPORT

A 26-year-old female had a history of a well-differentiated papillary thyroid cancer that was originally diagnosed when she was 16 years of age. At that time, the size of her tumor was 4 cm, and it was located within the right thyroid lobe. The patient underwent a subtotal thyroidectomy followed by adjuvant RAI therapy. Four years later at age 20 years, she developed a local recurrence that was treated with local re-excision only. Whether she received further therapy with RAI is not clear, as the records following this surgery were not available. When the patient was 26 years old, she was referred to our institution, presenting to the emergency department (ED) with intractable abdominal pain of several months’ duration, overall failure to thrive, and ongoing weight loss.

Review of her medical history revealed that she was noncompliant with taking levothyroxine, resulting in a thyroid-stimulating hormone (TSH) level of >100 mIU/L (normal range, 0.5-5.0 mIU/L) on initial presentation. Physical examination revealed a palpable, subcutaneous, partially movable, 1-cm nodule in the neck, just underneath the scar from her thyroidectomy. The patient also had midabdominal fullness with associated moderate pain on palpation and within the entire right subcostal region.

**Figure 1.**
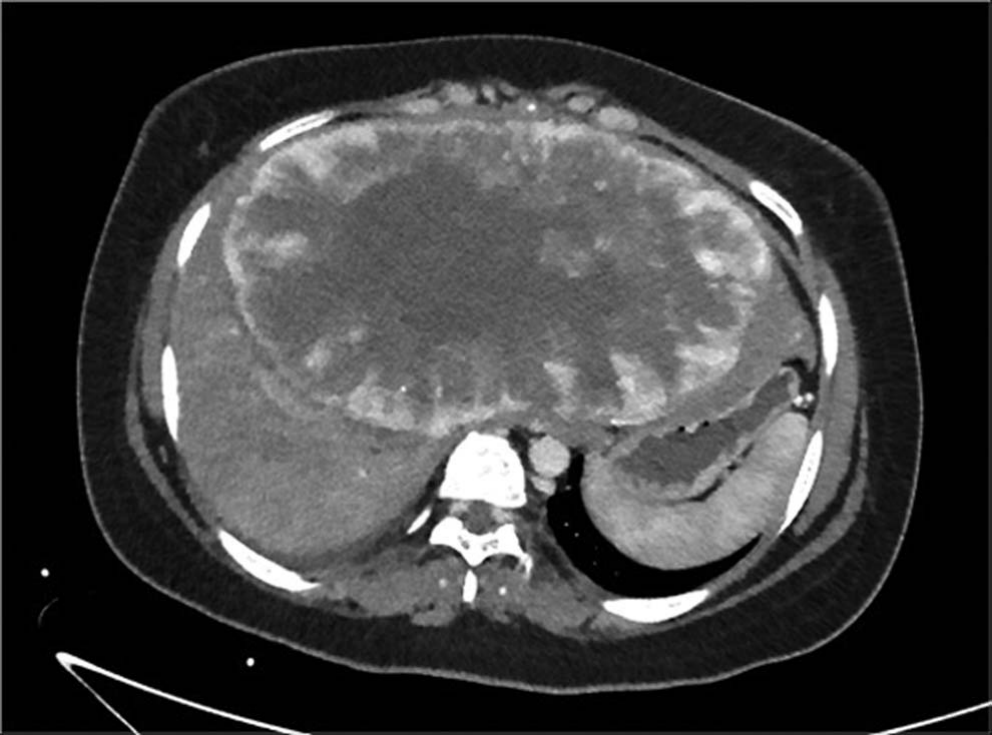
Computed tomography scan with contrast at the level of the upper abdomen shows a large (12 × 20 × 16 cm) heterogeneously enhancing mass centered in the left hepatic lobe and extending into the right lobe.

Computed tomography (CT) scan of the chest, abdomen, and pelvis revealed a large hepatic mass, measuring 12 × 20 × 16 cm in greatest dimension, occupying most of the central portion of the liver and extending into the right lobe (Figure 1). No other masses or lesions were identified. Further imaging studies with I-121 and I-131 revealed no significant uptake of iodine. Portal venogram confirmed a markedly elevated portal venous pressure of 21 mmHg (normal range, 5-10 mmHg), hepatic vein pressure of 18 mmHg (normal range, <5 mmHg), and normal right atrial pressure of 4 mmHg (normal range, 0-5 mmHg). The elevated pressures were attributed to both significant mass effect and compression of the hepatic veins from this large and bulky tumor mass.

**Figure 2.**
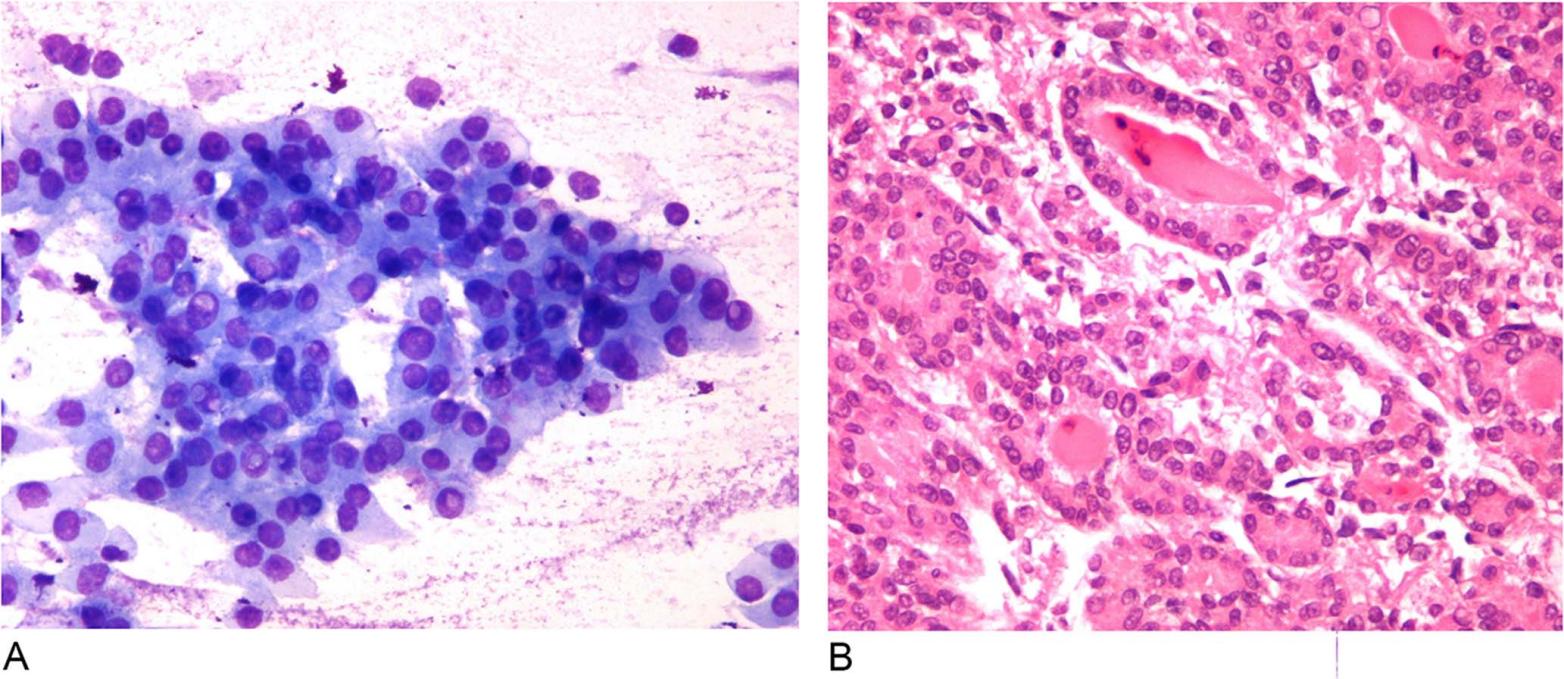
(A) Photomicrograph (×400) from fine needle aspiration biopsy of the thyroid nodule reveals cytologic features of papillary thyroid cancer characterized by groups of follicular cells with nuclear enlargement and intranuclear pseudo-inclusions. (B) Photomicrograph (×400) from core needle liver biopsy reveals metastatic papillary thyroid cancer exhibiting irregular, ovoid, crowded nuclei with nuclear pseudo-inclusions and follicular structures containing colloid and a multinucleated giant cell.

Final pathology from a fine needle aspiration biopsy of the neck nodule revealed cytologic features of papillary thyroid carcinoma, characterized by groups of follicular cells with nuclear enlargement and intranuclear pseudo-inclusions (Figure 2A). Surgical biopsy from the liver mass revealed metastatic papillary thyroid carcinoma, characterized by irregular, ovoid, crowded nuclei with nuclear pseudo-inclusions and occasional follicular structures containing colloid and multinucleated giant cells (Figure 2B). Immunohistochemical stains confirmed the diagnosis, with positive expression of thyroglobulin and thyroid transcription factor-1 within the tumor cells. Although an in-depth genomic analysis such as with next-generation sequencing could not be performed, we were able to confirm that the tumor did not contain a BRAF gene mutation, and fluorescence in situ hybridization analysis was negative for mutations of the NTRK1 gene.

We presented the patient's case at our multidisciplinary tumor board to discuss the available treatment options as the next steps in management. Given that treatment with RAI was not an option because of the limited uptake seen on I-121 and I-131 imaging, the consensus was to proceed with an initial open resection.

At the time of the operation, we mobilized the liver and identified an appropriate plane along the right lateral aspect of the tumor mass. Our attempts to adequately define the plane of resection resulted in a large amount of blood loss, mainly because of the marked hypervascularity of the mass, with multiple enlarged feeding veins extending into the tumor. Despite adequate intraoperative resuscitation of the patient, the tumor was determined to be unresectable, both because of the significant ongoing blood loss and the difficulty of adequately mobilizing the liver due to the large size and central location of the tumor. We also decided not to resect the neck nodule at this initial operation and to instead monitor the nodule for a possible response to systemic therapy.

After re-presentation of the case at our multidisciplinary tumor board, we decided that the next reasonable treatment option was to focus on a regional ablative approach. We chose locoregional therapy with selective internal radioembolization therapy (SIRT), using yttrium-90 (Y-90) microspheres (SIRT-Y-90), with the goal of shrinking the tumor size and reducing incoming blood flow to facilitate tumor resection at the second operation. Although other locoregional approaches were discussed, such as transarterial chemoembolization, the consensus was that the tumor was simply too large to expect an adequate reduction in the overall size.

**Figure 3.**
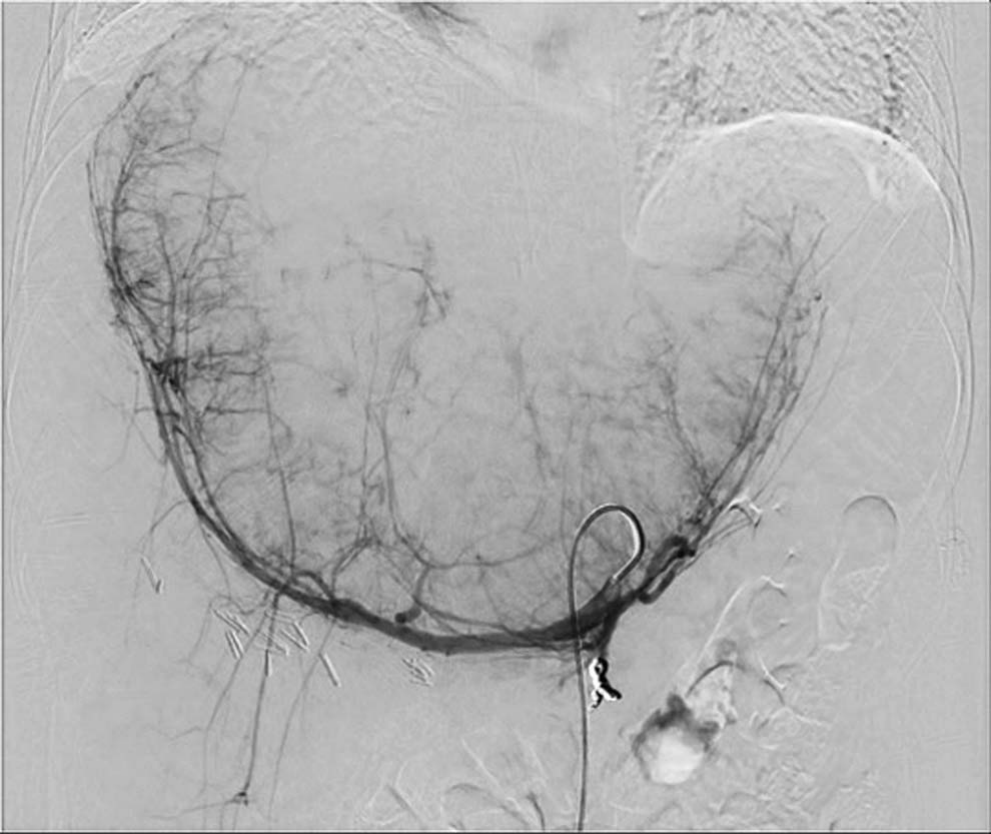
Angiogram performed after selective coil embolization of the gastroduodenal artery in preparation for yttrium-90 microsphere radioembolization delivery via the hepatic artery shows patchy contrast enhancement of the enlarged and hypervascular liver mass. Injection was performed through a catheter in the celiac artery.

Y-90 treatment began with an outpatient mapping angiogram to further evaluate perfusion of the tumor and liver and to calculate the lung shunt fraction. Angiograms showed the left and right gastric arteries were near the potential site of treatment, so coil embolization of these branches was performed to prevent nontarget embolization of radioactive particles that could potentially injure the stomach (Figure 3). Of note, the gastroduodenal artery was not visible, possibly because chronic compression or kinking of the artery led to collateral perfusion of this territory from the superior mesenteric artery. A small dose of radiation (5.2 mCi technetium-radiolabeled macroaggregated albumin) was injected into the hepatic artery to evaluate the site of embolization and ensure an acceptable level of shunting to the lung. Postprocedure nuclear scintigraphy showed that the test dose of radiation embolized the liver without embolizing the stomach or intestines, and the lung shunt fraction was low at 7.6%, which is below the safety threshold of 10% described in the package insert.

Two weeks after the angiogram was performed, the patient returned for SIRT-Y-90. SIR-Spheres Y-90 resin microspheres (Sirtex Medical) were chosen based on local practice, and a radiation dose of 2.2 GBq was calculated using the empirical dosimetry model. During the procedure, an angiographic catheter was inserted into the proper hepatic artery, and angiograms confirmed that the catheter tip was at the same location as the site of the test dose injection. No visible perfusion of tissues was visible other than the liver. SIR-Spheres Y-90 resin microspheres were injected through the catheter, leading to embolization of particles into the liver and tumor. After the procedure, nuclear scintigraphy showed a large dose of radiation within the tumor and no signs of nontarget radiation visualized. The patient was discharged later the same day without complication.

In parallel to the use of SIRT-Y-90 and as part of a multipronged approach to achieve maximal tumor shrinkage, we started the patient on systemic therapy with the oral tyrosine kinase inhibitor (TKI) lenvatinib, which is US Food and Drug Administration (FDA)–approved for the treatment of patients with locally recurrent or metastatic, progressive, RAI-refractory DTC. The total duration of the combined treatment with lenvatinib (24 mg orally, once daily) and levothyroxine (25 μg orally, once daily) was 6 months, with the levothyroxine reducing the patient's TSH level to the goal of <0.1 mIU/L. Physical examination of the neck mass revealed shrinkage of the overall size by approximately 50%.

**Figure 4.**
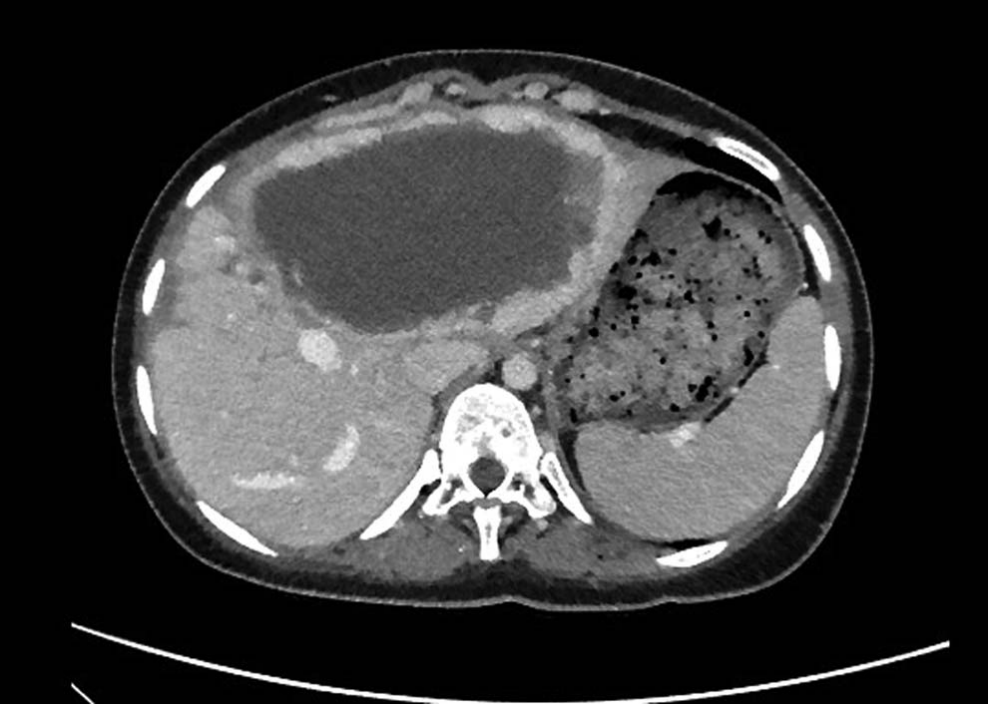
Computed tomography scan with contrast at the level of the upper abdomen performed 6 months after radioembolization with yttrium-90 treatment shows a tumor size of 8 × 15 × 14 cm, representing an approximately 67% overall reduction in tumor volume.

At this point, repeat CT scan of the abdomen and pelvis demonstrated marked central tumor necrosis associated with a significant overall diameter reduction in the metastatic tumor (Figure 4). The original mass in the liver measured 12 × 20 × 16 cm and was approximately 2,100 mL in volume; the comparative CT scan postprocedure showed shrinkage of the mass to 8 × 15 × 14 cm and approximately 700 mL in volume. Tumor volume reduction of 67% had been achieved in addition to reduced perfusion, hepatic vein congestion, and mass effect.

Given the patient's excellent response to the multimodal therapy, we returned to the operating room, hypothesizing that we had achieved the maximal response to treatment and intending to perform a definitive resection of the isolated metastatic lesion for potential cure. Upon entering the abdomen, we encountered dense fibrotic changes with an associated diffuse inflammatory response, presumably related to the SIRT-Y-90. Despite this unexpected finding, we successfully removed the entire metastatic lesion via extended left hepatectomy, leaving an adequate portion of healthy-appearing liver in the right hepatic lobe. The resection margins were negative on final pathology.

The patient recovered from her operation and continued to thrive while being closely followed. After 19 months, the patient began complaining of vague right flank pain that was confirmed to be associated with a fullness of the soft tissue overlying the intercostal region on physical examination. Imaging studies with a whole-body positron emission tomography/CT revealed a single hypermetabolic lesion involving the soft tissue and bony region of the right 7th rib. The only other hypermetabolic focus was the previously identified locally recurrent neck nodule, located in the subcutaneous tissue of the resection bed of the previous subtotal thyroidectomy. We agreed on definitive surgical resection of both areas and returned to the operating room soon after. The final pathology for both lesions showed findings of papillary thyroid carcinoma.

Postoperatively, we recommended that the patient continue levothyroxine treatment to maintain a suppressed TSH level. However, after being lost to follow-up for approximately 14 months, she re-presented to the ED with a TSH level of 62 mIU/L and new, bony metastatic disease to the thoracic spine and left femur. The patient had been noncompliant with her recommended treatment regimen throughout the 14 months. She was restarted on levothyroxine and lenvatinib therapy, but progression of metastatic disease continued. The patient was offered several clinical trial opportunities but elected to enroll in a home hospice program and died soon after. From the time of her original diagnosis of metastatic disease until her death at age 29 years, the total time of the patient's clinical course and treatment was approximately 39 months.

## DISCUSSION

This complex case highlights numerous care issues related to sequencing therapies and the optimal approach to managing patients with RAI-refractory, metastatic DTC. Our initial approach—attempted surgical resection—was likely overly ambitious as we discovered intraoperatively that the metastatic lesion was too large and vascularized to safely resect. We recognized the problem early during the operation and were able to control ongoing blood loss, adequately resuscitate the patient, and allow for her postoperative recovery. Our mindset following this operation was “to return and fight another day,” hoping to have a future opportunity for definitive surgical resection if we could effectively shrink the tumor by at least 50% with nonoperative approaches. The entire cancer care team discussed all possible alternative treatment options that would maximize the possibility of tumor cytoreduction and shrinkage. The clear consensus among the team was to proceed with a multimodal, neoadjuvant approach to treatment, settling first on SIRT-Y-90, concomitant with systemic therapy using an oral TKI.

Radioembolization with Y-90 was successfully performed in the outpatient setting without complications, and the treatment was well tolerated by the patient. The safety of SIRT-Y-90 in the treatment of various types of nonthyroid liver metastases is well established.^15^ Additionally, SIRT-Y-90 is effective in downstaging both primary and secondary (metastatic) hepatic malignancies, such as neuroendocrine tumors and metastatic colon and breast cancer.^16^ Bester et al used SIRT-Y-90 to safely and effectively aid in the local control of unresectable liver metastases, especially in the salvage setting.^17^

To our knowledge, this case is the first documented report of a patient with an isolated metastatic liver lesion of DTC that was treated with SIRT-Y-90 with or without adjunctive oral TKI therapy. We were unable to find a case report or a series of patients that described a similar case in which SIRT-Y-90 was used for both locoregional control and as an adjunct to definitive surgical resection.

Immediately following the successful SIRT-Y-90, we discussed systemic treatment options that could provide additional benefit toward our goal of maximizing tumor shrinkage and minimizing the development of extrahepatic metastatic disease, with a specific focus on sorafenib and lenvatinib. Both FDA-approved, multitargeted TKIs have been shown to be efficacious for the treatment of RAI-refractory thyroid cancer. Sorafenib was the first TKI to show an improvement in progression-free survival (PFS) but not overall survival when compared to a placebo control arm, with a median PFS of 10.8 vs 5.8 months, a statistically significant difference of 5 months.^18^ Of note, the response rates were 12.2% in the sorafenib arm, and all cases were noted to have a partial response, with the median overall survival not reached for this study. The development of resistance to sorafenib treatment after 12 to 24 months is almost a certainty with prolonged treatment, so secondary treatment options such as lenvatinib may be necessary.^19^

Lenvatinib is a more recently developed oral multitargeted TKI of vascular endothelial growth factor receptors 1, 2, and 3; fibroblast growth factor receptors 1 through 4; platelet-derived growth factor receptor-α; and RET and KIT signaling networks.^20^ A phase-3, double-blind, randomized multicenter study—the SELECT trial—compared treatment with lenvatinib vs placebo in patients with RAI-refractory thyroid cancer.^21^ The group treated with lenvatinib had a highly significant PFS advantage of 18.3 vs 3.6 months in the placebo group. The response rate was 64.8% in the lenvatinib group, with 4 complete responses and 165 partial responses, compared to a 1.5% response rate in the placebo group. Our decision to begin therapy with lenvatinib instead of sorafenib was based on the much higher response rates with lenvatinib and the lower likelihood of developing resistance with extended therapy. We were also hoping for a synergistic response with the previous SIRT-Y-90 treatment to maximize tumor shrinkage.

Another important clinical consideration in our approach to treatment was the active suppression of TSH levels, considered a vitally important component of therapy for recurrent DTC. Our patient's case was likely complicated by her apparent noncompliance with her prescribed levothyroxine therapy. We immediately started the patient on supratherapeutic doses of levothyroxine to maintain a goal TSH level of <0.1 mIU/L, which was the recommendation for patients with active thyroid carcinoma or for patients at a high risk for recurrence. In retrospect, this patient likely had metastatic disease beyond the liver and involving the bone at her initial presentation, even though the disease was not visualized with standard imaging modalities.

## CONCLUSION

We treated this case of a solitary metastatic DTC to the liver with a multidisciplinary approach involving both surgical and nonsurgical therapies and reduced the size of the tumor by approximately 67%. The marked reduction in tumor mass and diameter after neoadjuvant SIRT-Y-90 therapy combined with systemic lenvatinib allowed us to definitively resect the tumor with negative surgical margins on final pathology. This case highlights the importance of a team approach to discuss all possible treatment options, as well as the correct sequencing of each therapy to maximize the goals of treatment.

## Notes

*This article meets the Accreditation Council for Graduate Medical Education and the American Board of Medical Specialties Maintenance of Certification competencies for Patient Care and Medical Knowledge.*
